# Ultrastructural Analysis of *Candida albicans* When Exposed to Silver Nanoparticles

**DOI:** 10.1371/journal.pone.0108876

**Published:** 2014-10-07

**Authors:** Roberto Vazquez-Muñoz, Miguel Avalos-Borja, Ernestina Castro-Longoria

**Affiliations:** 1 Departamento de Microbiología, Centro de Investigación Científica y de Educación Superior de Ensenada (CICESE), Ensenada, B.C., Mexico; 2 División de Materiales Avanzados, Instituto Potosino de Investigación Científica y Tecnológica (IPICYT), San Luis Potosí, S.L.P., México; 3 Centro de Nanociencias y Nanotecnología, Universidad Nacional Autónoma de México (UNAM), Ensenada, B.C., Mexico; Universidade Federal de Juiz de Fora, Brazil

## Abstract

*Candida albicans* is the most common fungal pathogen in humans, and recently some studies have reported the antifungal activity of silver nanoparticles (AgNPs) against some *Candida* species. However, ultrastructural analyses on the interaction of AgNPs with these microorganisms have not been reported. In this work we evaluated the effect of AgNPs on *C. albicans*, and the minimum inhibitory concentration (MIC) was found to have a fungicidal effect. The IC_50_ was also determined, and the use of AgNPs with fluconazole (FLC), a fungistatic drug, reduced cell proliferation. In order to understand how AgNPs interact with living cells, the ultrastructural distribution of AgNPs in this fungus was determined. Transmission electron microscopy (TEM) analysis revealed a high accumulation of AgNPs outside the cells but also smaller nanoparticles (NPs) localized throughout the cytoplasm. Energy dispersive spectroscopy (EDS) analysis confirmed the presence of intracellular silver. From our results it is assumed that AgNPs used in this study do not penetrate the cell, but instead release silver ions that infiltrate into the cell leading to the formation of NPs through reduction by organic compounds present in the cell wall and cytoplasm.

## Introduction

Fungal infections are among the leading causes of infectious diseases [1], [2], with *Candida* being the most representative model of pathogenic yeasts in humans [3], [4]. *Candida albicans* is a dimorphic fungus which is present as an important component of the normal flora in healthy people [5], but in conditions of a weakened immune system *Candida* becomes an opportunistic pathogen following a transition from a commensal to a pathogenic phase [6]. *Candida* species are able to form biofilms and are the primary cause of mortality in immunocompromised patients, as they cause invasive candidiasis, which is difficult to eradicate due to high resistance to antifungal treatments [7]. In fighting fungal infections, some antifungal substances have three main disadvantages: limited range of action, self-medication, which may interact negatively with different types of antifungal agents, and the resistance of microorganisms [8], [9]. Furthermore, despite improvement of antifungal therapies over the last 30 years, antifungal resistance is still of major concern in clinical practice [10], and in general, the development of new antibiotics is a long and expensive process [11] which can now be resolved with the development of nanomaterials exhibiting antibiotic properties. Silver has long been recognized as an effective antimicrobial agent even at low concentrations [12], and recently silver nanoparticles have gained recognition as a promising antibacterial/antifungal agent [13], [14]. Silver is used in clinics to treat pathogenic infections in skin wounds, burns, and transplant surgery [15]; however, chronic use of silver to treat diseases or taking colloidal silver as a dietary supplement could lead to potential toxic effects [16], [17], [18]. In fact, it is known that chronic ingestion of silver can cause argyrosis and argyria [17], [19] and while these are not life-threatening conditions, they are cosmetically undesirable [20]. Silver is eliminated from the human body by the liver and kidneys and apparently is not toxic at low concentrations [20], but at higher concentrations it is considered to be potentially toxic to human cells, inhibiting the synthesis of proteins and DNA [16]. Due to the importance of silver in health care, recent studies suggest that AgNPs may be safer to use than ionic or colloidal silver [21]–[23]. Recently, Huh and Kwon [23] defined the term “nanoantibiotics” as nanomaterials with antimicrobial activity and/or for those that enhance the effectiveness and safety of antibiotics, of which currently AgNPs are among the most studied [24]–[28]. Potential positive effects in using AgNPs are the prevention of biofilm formation [29], anti-inflammatory activity [30]–[31], antiviral capacity [32], and use in post-burn treatments [33]. It has also been reported that AgNPs have synergistic interactions with different antimicrobial drugs [34]–[36], which is particularly important because in some treatments, the amount of drug could be reduced along with AgNP concentration. However, more research is necessary to elucidate the safety of using AgNPs in the treatment of human diseases. In this respect the majority of reports currently aim at their use against pathogenic bacteria, but studies on the effect of AgNPs against other microorganisms such as fungi, protozoans, and viruses are limited. Furthermore, most studies related to AgNPs-biological systems are focused on the biocidal effect of nanomaterials on microbes, and only a limited number of studies explore the interactions and possible action mechanisms of AgNPs in microbial inhibition. Antifungal activity by AgNPs has been proved against different *Candida* species; *C. albicans*, *C. glabrata,* and *C. tropicalis* were completely inhibited using AgNPs [37]. Furthermore, it was reported that AgNPs damage the structure of the cell membrane in *C. albicans* producing “holes” on the surface of the cells and thus inhibiting the budding process [21]. Also, the production of reactive oxygen species (ROS), DNA fragmentation, and apoptosis has been reported [38]. Although the antifungal effect of AgNPs is generally known, their interaction with biological systems is not fully documented. Therefore, the objective of this work is to evaluate the effect of a commercial AgNPs product against *C. albicans* and to determine how AgNPs interact with *Candida* cells at the ultrastructural level; this information will complement existing information and could contribute to generating novel broad-spectrum antifungal treatments.

## Materials and Methods

### Strain, media and growth conditions

*Candida albicans* ATCC SC5314 strain was cultured at 37°C in liquid and solid media: YPD (1% yeast extract, 2% peptone, 2% dextrose) and YPD agar plates (2% bacteriological agar added). The culture media were prepared using distilled water and sterilized by conventional methods. AgNPs used in this work were from Vector Vita Ltd, Novosibirsk. The product is defined as a medicine “Argovit” which is clustered silver (AgNPs) functionalized with polyvinylpyrrolidone (PVP) and the reported average size of initial cluster particles is about 1.5–2 nm [39]. Silver concentration of the product is 12,000 µg/mL.

### Minimum inhibitory concentration (MIC)

The minimum inhibitory concentration (MIC) of AgNPs was determined by a macrodilution test as follows: Cells were grown on YPD agar plates for 24 hours, at 37°C. Then 2–10 colonies were inoculated in YPD broth and the cell density was adjusted using a spectrophotometer. Immediately, the cultures were exposed to the different treatments (AgNPs, FLC and AgNPs−FLC) and incubated at 37°C for 24 hours at 250 rpm (Orbit Environ Shaker). Cultures of three cell densities were exposed at different silver concentrations, and after treatment, the cells were inoculated into YPD agar plates and were allowed to incubate for 24 hours at 37°C. To determine the combined effect of AgNPs with a commercial antifungal product, using the method described above, the half–maximal inhibitory concentration (IC_50_) was determined for AgNPs and fluconazole (FLC). The standard growth curve of absorbance (abs) vs. cell density in YPD was performed at λ = 520 nm in a spectrophotometer Jenway 6505 UV-Vis; all measurements were done in triplicate, and standard deviation (σ) was calculated.

### Sample preparation for transmission electron microscopy (TEM)

For microscopic analysis, cultures exposed to the determined MIC of AgNPs were first observed after 24 hours of incubation under bright-field microscopy, using a Zeiss Axiovert 200 M. After that, cultures were prepared for transmission electron microscopy (TEM) analysis. During the preparation process, samples were centrifuged at each step of the protocol, 5 min at 1500 rpm for the fixation and dehydration processes and 10 min at 3000 rpm during infiltration. Obtained pellets were fixed with 2% glutaraldehyde in 0.05 M sodium phosphate for 30 minutes at room temperature. After fixation cells were washed with sodium phosphate and post fixed with 1% OsO_4_, for 2 hours at 4°C. Afterwards, samples were dehydrated in ethanol series (15, 30, 50, 75 and 100%) for 2 hours at each step. Then samples were infiltrated in resin/ethanol series (20/80, 40/60, 60/40, 80/20) for 3 hours at each step and left overnight at 100% Spurr’s resin. Finally the pellets were placed in coverslips previously coated with Teflon-like spray and “sandwiched” in 100% Spurr’s resin for polymerization at 60°C for 24 hours. After cooling, coverslips were separated and examined under a stereoscopic microscope to cut pieces of resin containing pellets. Polymerized pellets were mounted in resin blocks and sectioned in an ultra-microtome Leica Ultracut R. Thin sections of 70 nm were mounted in formvar/carbon 75 mesh copper grids and analyzed under TEM (Hitachi H7500 operated at 100 keV and spot size 5). Sections were examined without post-staining for better silver detection.

### High-resolution transmission electron microscopy (HRTEM)

To determine chemical characterization of *C. albicans* ultrathin sections and silver crystallographic analysis, samples previously scanned under TEM were examined under high-resolution transmission electron microscopy (HRTEM) (Tecnai F30 operated at 300 keV and spot size 6). Analyses included Scanning Transmission Electron Microscopy (STEM) and Energy Dispersive X-ray Spectroscopy (EDS).

## Results

**Figure 1:**
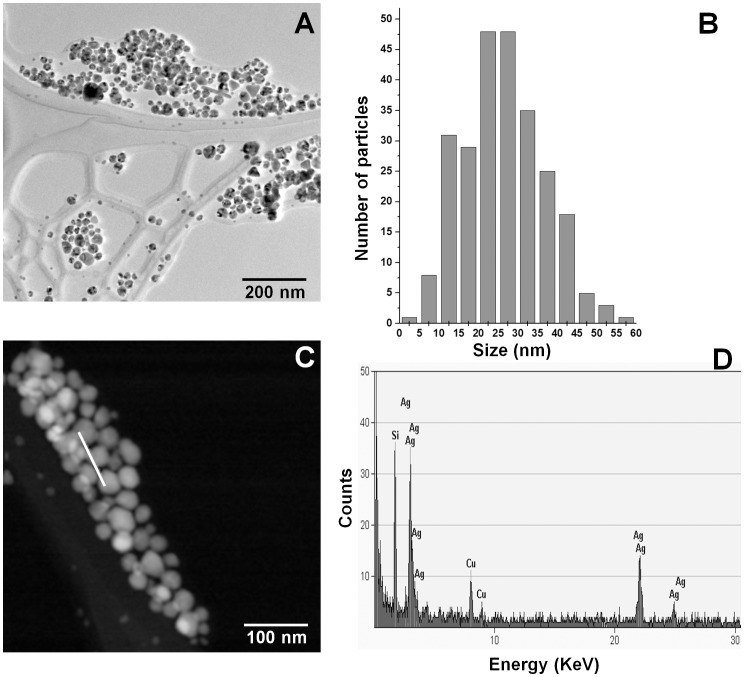
Characterization of silver nanoparticles (AgNPs) used in this study. A) Transmission electron micrograph of AgNPs functionalized with Polyvinylpyrrolidone (Vector-Vita Ltd., Russia), B) histogram of measured nanoparticles, C) High-angle annular dark-field imaging (HAADF) of AgNPs, D) Typical EDS analysis of particles along the trace indicated by a white line in (C).

It is known that AgNPs are highly unstable, and previous studies have shown that size of AgNPs could influence their antimicrobial activity. Therefore, the NPs used to treat *C. albicans* were examined under TEM to determine shape and size range at the moment of the experimental procedure. It was found that AgNPs were mainly quasi-spherical, although other shapes were also detected (Figure 1A). Size range was 3–60 nm (Figure 1B) before and after application, being different from that reported by the authors [39]. The elemental character of nanoparticles was also determined and the EDS analysis confirmed the presence of silver (Figure 1C–D). The presence of copper in the results obtained could be due to the grid we used; however, the signal of silicon (Figure 1D) could be a trace component of the commercial product we used.

**Figure 2:**
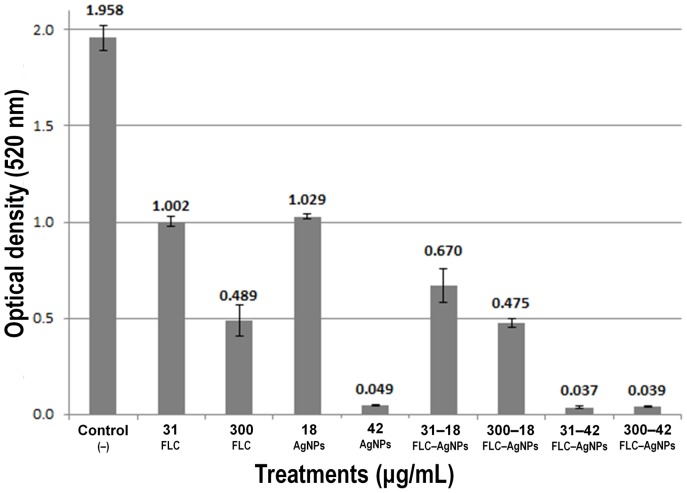
The combined effect of silver nanoparticles (AgNPs) and fluconazole (FLC) in *C. albicans* reduces cell proliferation. Liquid cultures were exposed to the IC_50_ of FLC and several combinations of AgNPs-FLC.

**Figure 3:**
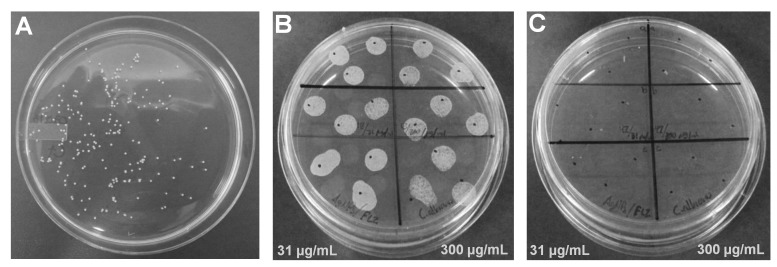
Subcultures of *C. albicans* in YPD agar plates after 24 h of incubation. A) Representative image of a culture to determine the IC_50_ of AgNPs and FLC; B) subcultures of the combined effect of the IC_50_ of AgNPs (18 µg/mL) and two concentrations of FLC; C) subcultures of the combined effect of the MIC of AgNPs (42 µg/mL) and two concentrations of FLC. In B–C the IC_50_ of FLC (31 µg/mL) was used in the inoculations on the left side of the plate and 300 µg/mL of FLC on the right side.

The minimum inhibitory concentration (MIC) of AgNPs depends greatly on the initial inoculum and the size/shape of NPs; therefore, the MIC was determined for cultures of different cell densities (Table 1). In all cases the MIC was equivalent to the minimum fungicidal concentration (MFC). After 24 h of exposure to treatments, samples were re-inoculated in YPD agar plates, and after 24 h of incubation no growth was detected in cultures exposed to AgNPs. Also, since the combined use of an antimicrobial drug with AgNPs reduces the amount of the drug used to inhibit microorganisms, the effectiveness of the combined effect of AgNPs with a fungistatic drug was determined; we used the IC_50_ of fluconazole (FLC) and the IC_50_ of AgNPs against different *Candida* cell concentrations. Using 2.5×10^6^ cells/mL, the IC_50_ of AgNPs was 60 µg/mL and 125 µg/mL for FLC; for 1×10^4^ cells/mL the IC_50_ was 18 and 31 µg/mL for AgNPs and FLC, respectively (Table 2). After 24 h of incubation using the combined IC_50_ of AgNPs−FLC against *Candida* (1×10^4^ cells/mL), the optical density of the culture was lower than that of the control and the culture exposed only to the IC_50_ of FLC (Figure 2). The optical density using an increased concentration of FLC (10× IC_50_ = 300 µg/mL) with the same IC_50_ of AgNPs was similar to the use of 300 µg/mL of FLC alone (Figure 2). When the MIC of AgNPs was used (42 µg/mL) in combination with the IC_50_ of FLC (31 µg/mL) and with 300 µg/mL of FLC, *Candida* was completely inhibited (Figure 2). The optical densities recorded using those concentrations were due to the absorbance of AgNPs. To determine cell viability, samples were inoculated in YPD agar plates and growth was not recorded using 42 µg/mL of silver concentration (Figure 3).

**Table 1:** Minimum inhibitory concentration (MIC) of AgNPs for *C. albicans*, at different cell concentrations.

Initial inoculum and optical density (D.O) of the culture (λ = 520)	AgNPs (µg/mL of silver)
4.3×10^7^ cells/mL, O.D = 0.706	600
2.5×10^6^ cells/mL, O.D. = 0.082	150
1×10^4^ cells/mL, O.D. = 0.040	42

**Table 2:** Cell density and concentrations of AgNPs and FLC used to determine the half–maximal inhibitory concentration (IC_50_) of fluconazole (FLC) and AgNPs (silver) to inhibit *C. albicans.*

*C. albicans*	Treatment	Concentration (µg/mL)
2.5×10^6^ cells/mL	AgNPs	150, 120, 90, **60**, 42, 18
	FLC	300, **125**, 63, 31,15
1×10^4^ cells/mL	AgNPs	90, 60, 42, **18**, 6
	FLC	125, 63, **31**, 15, 7

The IC_50_ for each cell concentration is underlined.

Control cultures and cultures exposed to equivalent concentrations of the stabilizing agent polyvinylpyrrolidone (PVP) were also incubated at 37°C. It was determined that PVP does not exert inhibitory effects on *Candida* cells, as there was no difference between the growth of cultures treated with PVP (abs = 2.207, σ = 0.042) compared to the control cultures (abs = 2.203, σ = 0.055). Growth in YPD agar plates media was also similar in both cases (images not shown).

**Figure 4:**
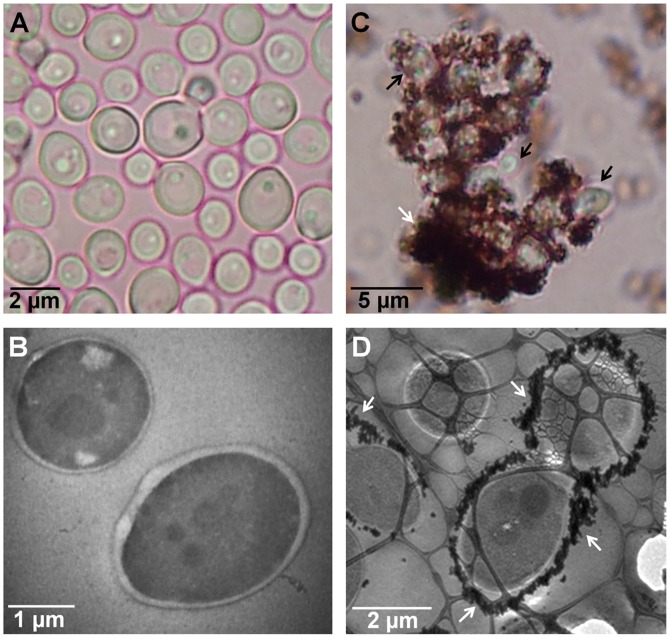
Microscopic analysis of *C. albicans* from liquid cultures. A, B) Cells from control cultures observed under optical bright field microscopy and TEM, respectively; C, D) Cells exposed to silver nanoparticles were agglomerated and surrounded by AgNPs as seen by optical bright-field microscopy (C) and confirmed by TEM (D). Black arrows indicate *Candida* cells and white arrows indicate AgNPs aggregation.

**Figure 5:**
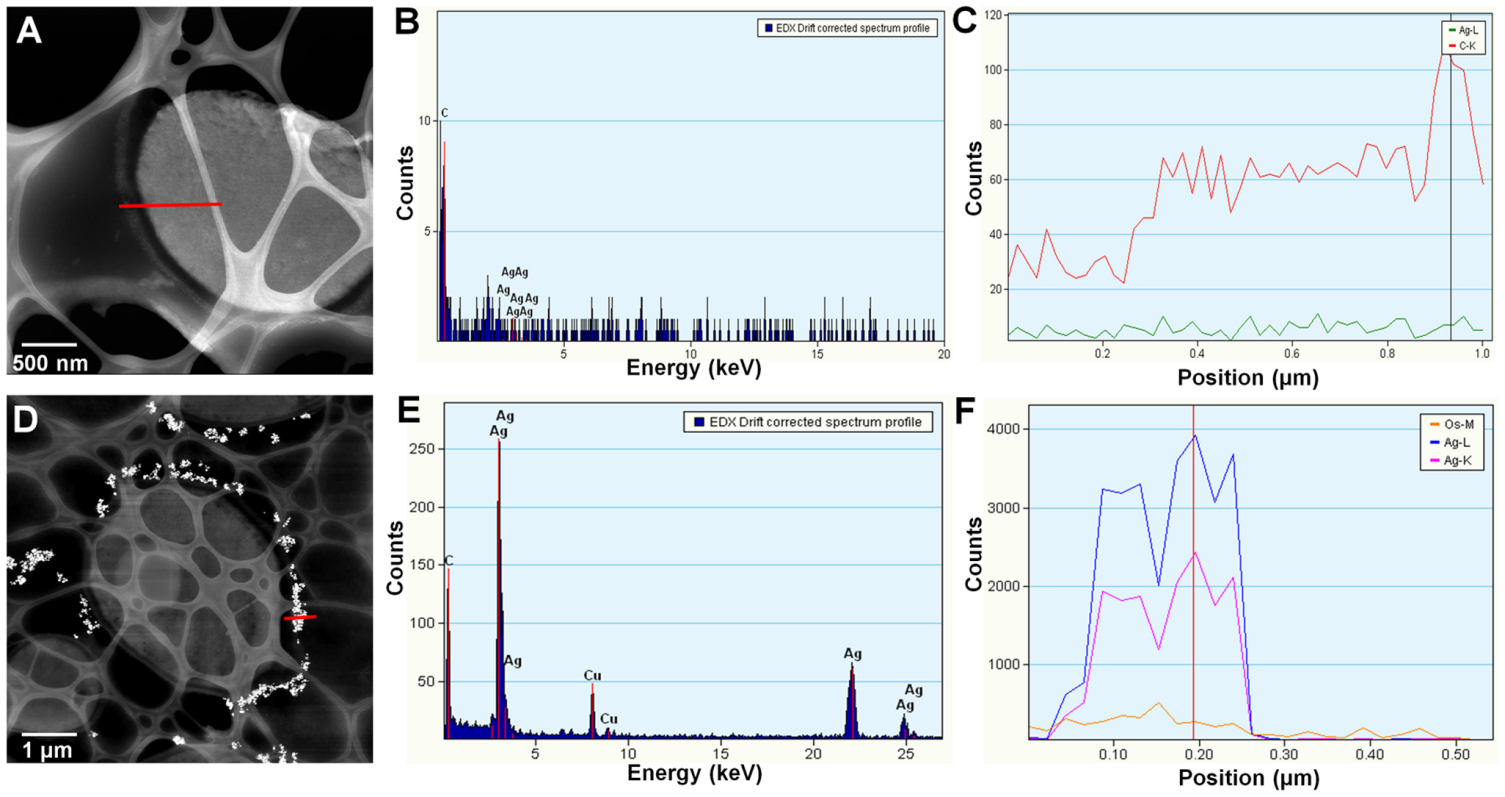
Chemical characterization of *C. albicans* ultrathin sections. A, D) High-angle annular dark-field imaging (HAADF) of analyzed cells; (B, E) EDS analysis showing the absence of silver in (A) and the presence of silver in (D); (C, F) Lineal EDS spectrum of (A) and (D), respectively. Red line in A and D indicates the transect where chemical analysis was performed.

**Figure 6:**
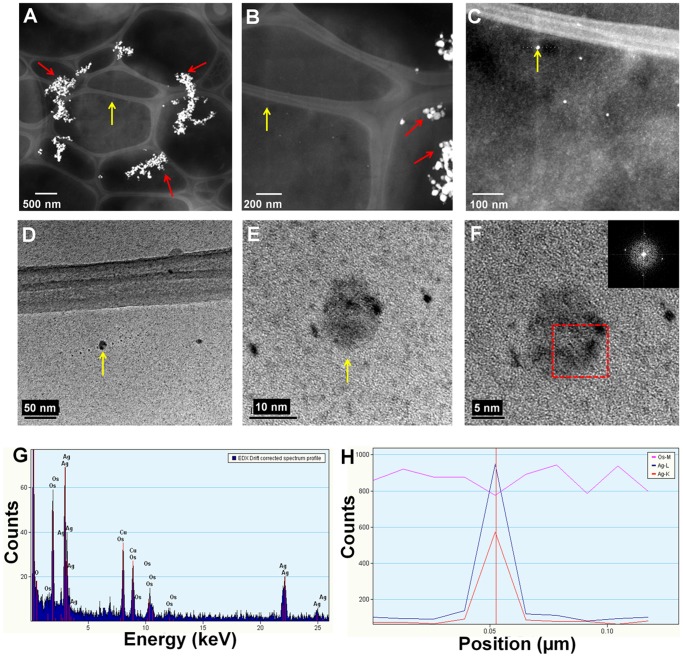
Chemical characterization of intracellular nanoparticles. A) HAADF image in which analysis of internal AgNPs was carried out, B) Closer view of internal-external particles, C) Amplified image of analyzed internal particle, (D–F) Images of analyzed internal particle, G) EDS analysis showing the presence of silver, H) Variation of Ag and Os along a trace line at neighbor points near the particle indicated by a yellow arrow in Figs A to E; sampled points can be seen as black dots in D and E. Yellow arrows point out analyzed particle, red arrows point out extracellular AgNPs. Enclosed area in (F) indicates the zone where chemical analysis was conducted, and the image in the upper corner is the diffraction pattern of analyzed particle, confirming the presence of crystalline silver.

**Figure 7:**
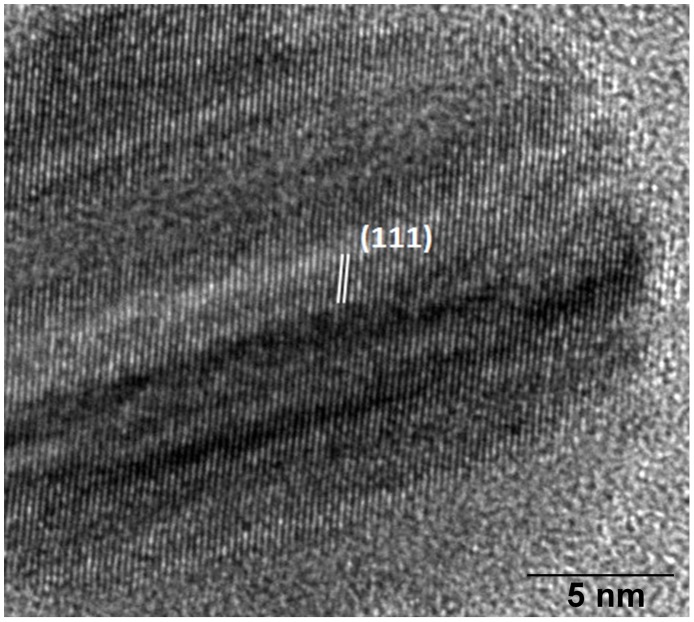
Crystallographic analysis of intracellular nanoparticles in *C. albicans*. Silver nanoparticle shows (111) planes with 0.24 nm spacing.

**Figure 8:**
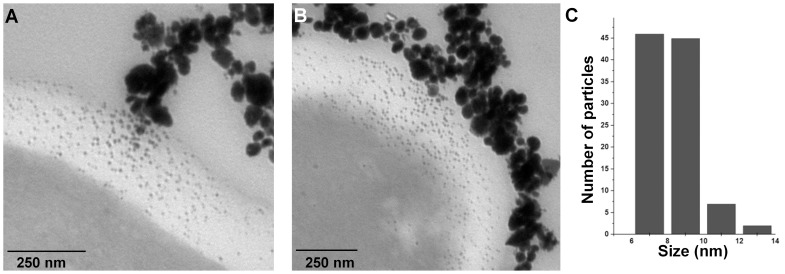
TEM images showing interaction of silver nanoparticles with *C. albicans*. A, B) Sections of cells in which extracellular agglomeration of AgNPs coincided with the accumulation of smaller AgNPs in the cell wall and cytoplasm, C) Size distribution of intracellular AgNPs.

Using bright field microscopy, cultures were first examined to determine cell morphology. Normal cell growth was observed in control cultures (Figure 4A) while in those treated with AgNPs cells were observed agglomerated, with nanoparticles surrounding *C. albicans* cells (Figure 4B). TEM analysis revealed that cells in control cultures and cells exposed to AgNPs did not show severe damage, such as the formation of pits or the rupture of the cell wall (Figure 4B, D). On the other hand, AgNPs were found in most cases surrounding the examined cells (Figure 4D). Chemical analysis was carried out throughout a zone including the cell wall and part of the interior of the cells (Figure 5A, D). Samples were examined to determine the elemental character of particles surrounding the cells. No silver was found in the control samples. Only noise was detected (Figure 5B, C), while in cells exposed to AgNPs, the presence of silver was clearly confirmed (Figure 5E, F). A closer inspection in the cytoplasmic area revealed small dots of approximately 10 nm in size (Figure 6A–F); therefore, chemical analysis was performed to corroborate the nature of those NPs. EDS analysis confirmed the presence of silver (Figure 6G–H), and crystallographic analysis using HRTEM confirmed the occurrence of intracellular silver crystals (Figure 7). TEM analyses clearly showed that close to the extracellular accumulation of AgNPs, a high accumulation of very small particles was present in the cell wall and part of the cytoplasm (Figure 8A–B). The size of those particles was in the range of 6–12 nm in diameter (Figure 8C), although other sizes were also present in the cytoplasm. It is interesting to note that in Figure 8B the border between cytoplasm and cell wall is not clearly defined as in Figure 8A, so it may be possible that AgNPs disrupt these structures and may cause severe damage at higher exposure times.

## Discussion

According with the definition of fungistatic versus fungicidal effect [40], in our study AgNPs were found to present a fungicidal effect, since cultures treated with the MIC were unable to recover after treatment, even after inoculation in fresh culture medium in successive subcultures. The antifungal properties of AgNPs against *C. albicans* have been demonstrated in some other studies, although reported MIC values are different from the ones we found in this work [21], [24], [28], [37], [38], [41], [42]. Such differences could be due to the nature of the particles used, the difference in size being particularly important. It is known that size and shape of metallic nanoparticles influence their chemical, optical, and thermal properties [43]. Therefore, the size and other important characteristics could also modify the antimicrobial properties of nanoparticles. For instance, the bactericidal properties of differently shaped AgNPs against *E. coli* were found to be shape dependent [44], and size-dependent toxicity was reported in alveolar macrophages [45]. The stabilizing agent may also influence the antimicrobial activity of NPs, since lower MIC of stabilized AgNPs was used against *Candida* spp, when comparing antifungal activity with non-stabilized AgNPs [37]. In our study AgNPs are functionalized with PVP, which is considered biocompatible [46] and did not exert inhibition in *Candida*. It is important to mention that in our study, AgNPs (at concentration higher than 5 µg/mL) were observed to slowly sediment in YPD broth. The sedimentation was observed in the form of particle precipitation at the bottom of the test tubes while sedimentation in distilled water was not observed. The sedimentation could affect the effectiveness of AgNPs; therefore, this is another factor that should be evaluated when exposing microorganisms in different liquid culture media conditions.

We explored the synergistic effect of AgNPs−FLC against *Candida* and found that the combination of both agents significantly reduced cell viability, similar to a previous study’s findings [47]. The use of AgNPs in combination with antimicrobial drugs may be beneficial in the clinic, since the amount of both agents can be substantially reduced and thus avoid adverse effects caused by some drugs and the potential toxic effects to the chronic exposure to silver. Some studies have reported that AgNPs exhibit synergistic effects with antibacterial drugs such as amoxicillin [34], ampicillin, kanamycin, erythromycin, and chloramphenicol [36], penicillin G, clindamycin and vancomycin [35]. However, antagonistic interactions were detected with amoxicillin and oxacillin in a methicillin-resistant *Staphylococcus aureus* strain [48] and with chloramphenicol in *Pseudomonas aeruginosa* [31]. Studies on the combined effect of AgNPs with fungicidal drugs may be particularly useful since we used FLC, which is considered among the fungistatic agents. Moreover, it is known that even fungicidal agents have limited action in immunosuppressed patients [40]; therefore, a synergistic effect of AgNPs−fungicidal drug may be particularly helpful in some clinical cases.

Antimicrobial mechanisms of nanomaterials are not fully understood, but it is proposed that when they come into contact with cells, they provoke the production of reactive oxygen species (ROS), cell membrane disruption, mitochondrial damage, and DNA damage, among others [23]. Ultra-structural studies on the microbial interaction with nanostructured material are scarce; this information could provide a better understanding of the acting mode of NPs. There are several reports at the ultrastructural level on the interaction of AgNPs with bacteria; however, some of them present low magnification images making difficult to elucidate the localization of NPs. Nevertheless, the effectiveness of AgNPs against bacteria is clearly demonstrated, and in fact, AgNPs were shown to be effective against *E. coli*, with cells showing formation of “pits” in the cell wall. The silver nanoparticles were found to accumulate in the bacterial membrane, and some of them were reported to successfully penetrate into the cells [49]. Similar results were found in *E. coli* and *V. cholera*; it was established that AgNPs provoked changes mainly in the cell membrane morphology, producing a significant increase in their permeability, thus affecting the proper transport through the plasma membrane, resulting eventually in cell death. They also reported that silver NPs with small diameters penetrated into the cells [13].

As previously mentioned, the effect of AgNPs on fungal species is scarce, but the MIC to inhibit *Candida* spp has been reported by several authors. Nevertheless, it becomes necessary to elucidate how AgNPs interact with fungal cells in order to generate information that could be useful to develop new clinical techniques for AgNPs applications. The effect and possible mechanisms of AgNPs in *C. albicans* were recently reported as the production of ROS and nuclear fragmentation that leads to cell death [38]. However, as far as we are aware, there are very few studies that report at the ultrastructural level the effect of AgNPs with fungal cells. It was stated that exposure of *C. Albicans* to AgNPs produced significant changes to the membrane, the formation of “pits” on the cell surface, and finally the formation of pores and cell death [21]. In fact, they presented TEM micrographs, but locating NPs outside or inside the cell is not possible due to low magnification images. In our study, Ag NPs were found surrounding *C. albicans* cells, similar to the results found in bacteria [13], [48]; also, AgNPs were found non-specifically distributed within the cell cytoplasm and in different regions of the cell wall, but no severe damage was observed in the structure, which differs from a study reporting the presence of “holes” in the cell wall of *Candida* when treated with AgNPs [21]. Another study reports disruption of the cell wall and cytoplasmic membrane in *Cryptococcus neoformans* treated with AgNPs at an exposure time of 72 hours [50]. From our TEM analysis it was clear that the accumulation of small AgNPs in the cell wall coincided with the accumulation of extracellular NPs, which strongly suggests a dynamic release of silver ions (Ag+) by adjacent AgNPs which actively penetrate the cell and lead to intracellular biosynthesis of AgNPs. The results of HRTEM analysis of intracellular NPs show a set of lines spaced by 0.24 nm, which is consistent with (111) planes of silver (Figure 7). The gradual release of Ag+ by AgNPs has previously been suggested [51] so the internalization of Ag+ into the cell could have special relevance, as they may act as a reservoir increasing the duration of the antimicrobial effects. This is of primordial importance since AgNPs are demonstrated to inhibit the biofilm formation [29], thus preventing further and stronger infections, or they could represent increased durability of medical devices with AgNP contents. However, despite the clear antimicrobial properties of AgNPs, their potential use in the clinic should be carefully evaluated since there is a lack of basic knowledge on the potentially different antimicrobial properties of AgNPs which may vary depending on many factors, including the method of synthesis, size, shape, functionalizing agent, application method etc., and also their interaction in more complex systems such as plants, animals, and humans.

## Conclusions

The results obtained in this study complement existing research on the potential use of nanomaterials in biomedicine. The fungicidal capacity of AgNPs functionalized with PVP was determined. It was clearly demonstrated that although no dramatic damage to the fungal cells was observed, at least after the exposure time of 24 h, no cell viability was recorded. Another important result was to discover that the mode of action of AgNPs is to aggregate outside the fungal cells, releasing silver ions and thus inducing cell death through the reduction process resulting from the interaction of cell components with ionic silver.
